# Photoperiodic diapause under the control of circadian clock genes in an insect

**DOI:** 10.1186/1741-7007-8-116

**Published:** 2010-09-03

**Authors:** Tomoko Ikeno, Shinichi I Tanaka, Hideharu Numata, Shin G Goto

**Affiliations:** 1 Graduate School of Science, Osaka City University, Sugimoto, Sumiyoshi, Osaka 558-8585, Japan; 2 Graduate School of Science, Kyoto University, Sakyo, Kyoto 606-8502, Japan

## Abstract

**Background:**

Most organisms have evolved a circadian clock in order to anticipate daily environmental changes and many of these organisms are also capable of sophisticated measurement of daylength (photoperiodism) that is used to regulate seasonal events such as diapause, migration and polymorphism. It has been generally accepted that the same elements are involved in both circadian (daily) and seasonal (annual) rhythms because both rely upon daily light-dark cycles. However, as reasonable as this sounds, there remains no conclusive evidence of such a molecular machinery in insects. We have approached this issue by using RNA interference (RNAi) in *Riptortus pedestris*.

**Results:**

The cuticle deposition rhythm exhibited the major properties of circadian rhythms, indicating that the rhythm is regulated by a circadian clock. RNAi directed against the circadian clock genes of *period *and *cycle*, which are negative and positive regulators in the circadian clock, respectively, disrupted the cuticle deposition rhythm and distinct cuticle layers were produced by these RNAi. Simultaneously, *period *RNAi caused the insect to avert diapause under a diapause-inducing photoperiod whereas *cycle *RNAi induced diapause under a diapause-averting photoperiod. The expression patterns of juvenile hormone-regulated genes and the application of juvenile hormone analogue suggested that neither ovarian development itself nor a downstream cascade of juvenile hormone secretion, were disturbed by *period *and *cycle *RNAi.

**Conclusions:**

This study revealed that the circadian clock genes are crucial not only for daily rhythms but also for photoperiodic diapause. RNAi directed against *period *and *cycle *had opposite effects not only in the circadian cuticle deposition rhythm but also in the photoperiodic diapause. These RNAi also had opposite effects on juvenile hormone-regulated gene expression. It is still possible that the circadian clock genes pleiotropically affect ovarian development but, based on these results, we suggest that the circadian clock operated by the circadian clock genes, *period *and *cycle*, governs seasonal timing as well as the daily rhythms.

See Commentary: http://www.biomedcentral.com/1741-7007/8/115

## Background

The biosphere is affected by two predominant environmental rhythms - the daily cycle caused by the Earth's rotation about its own axis and the annual cycle of the seasons caused by the Earth's rotation about the Sun. All eukaryotes, and some prokaryotes, have evolved a circadian clock that is set by light to time various daily activities at the biochemical, physiological and behavioural levels. Some of these organisms have evolved photoperiodism, a response to the length of day or night for the timing of development, reproduction and diapause in anticipation of seasonal changes in the environment [1,2]. Bünning [3] first proposed functional involvement of the circadian clock into the photoperiodic clock for measuring the length of day or night. Sophisticated experimental designs, including night interruption as well as Bünsow and Nanda-Hamner protocols, verified the involvement of the circadian clock into photoperiodism in various organisms [4-8]. Since both circadian rhythms and photoperiodism rely upon daily cycles of environmental change, it seems reasonable to assume that the same clock elements are involved in both processes. However, the functional molecular elements involved in the photoperiodic response are still veiled.

In many organisms, interlocked negative feedback loops consisting of circadian clock genes underlie the circadian clock [9-11]. In the central clock residing in the brain of *Drosophila melanogaster*, the CYCLE (CYC)/CLOCK (CLK) heterodimer acts as a positive regulator of the transcription of *period *(*per*), *timeless *(*tim*) and other output genes, whereas the PER/TIM heterodimer acts as a negative regulator of CYC/CLK activity. Entraining this oscillator to light depends on CRYPTOCHROME, which is a blue-light photopigment that promotes TIM degradation when stimulated by light. The circadian clock is also distributed in many peripheral tissues [12]. Although some diversity in the molecular clockwork has been presented between the central and the peripheral clocks [13] and among insects [14], the functional roles of PER and CYC are considered to be identical - PER acts as a negative regulator and CYC acts as a positive regulator [9].

Several authors have pointed out the functional involvement of individual circadian clock genes in insect photoperiodic diapause [15-17]. However, these results could be due to the pleiotropic effects of these individual clock genes on diapause itself and may not involve the circadian clock as an integrated physiological function [18-20]. Thus, involvement of the circadian clock into photoperiodism has yet to be verified at the molecular level.

We approached this issue by using RNA interference (RNAi) in the bean bug *Riptortus pedestris *(Insecta: Heteroptera: Alydidae), formerly known as *R. clavatus*. *R. pedestris *exhibits a clear photoperiodic response, that is, its ovarian development is induced under long-day conditions but is suppressed under short-day conditions (diapause) [21] and the physiological mechanisms underlying the response have been extensively studied [22]. It should be noted that photoperiodic sensitivity persists even after adult emergence in this species [23]. *R. pedestris *also exhibits circadian rhythmicity in oviposition and diel patterns of feeding and locomotor activity [24-26]. However, these rhythms can be easily disturbed by manipulation. Thus, in the present study, we focused on the cuticle deposition rhythm.

The cuticle of insect exoskeletons is generally composed of an epi-, exo- and endocuticle. After adult emergence, the endocuticle thickens by alternating the deposition of two types of layers with different orientations of chitin microfibrils. This arrangement enhances the physical toughness of the exoskeleton: in lamellate layers, the microfibrils are secreted helicoidally from the epidermal cells, whereas in non-lamellate layers they are secreted unidirectionally. It has been verified that in some species the cuticle deposition rhythm is governed by a circadian clock residing in the epidermis [27,28]. These lamellate and non-lamellate cuticle layers can be observed between crossed polarizers under a microscope as bright and dark layers, respectively [29].

Here we demonstrate that the cuticle deposition rhythm in *R. pedestris *exhibited major properties of circadian rhythms. This suggests that the rhythm is regulated by a circadian clock. RNAi directed against *per *and *cyc *disrupted the cuticle deposition rhythm, indicating that *per *and *cyc *are core components of the circadian clock regulating the cuticle deposition rhythm. RNAi of *per *resulted in the deposition of a single dark layer, whereas that of *cyc *resulted in a single bright layer. This indicates that *per *and *cyc *RNAi arrests the circadian clock in distinct phases. The photoperiodic response was also disrupted by RNAi of the circadian clock genes: *per *RNAi induced ovarian development even under short-day conditions, whereas *cyc *RNAi suppressed ovarian development even under long-day conditions. Thus, circadian clock genes are crucial, not only for daily rhythms but also for seasonal adaptation. Although it is also possible that these clock genes pleiotropically regulate the ovarian development, distinct effects of *per *and *cyc *RNAi on both circadian rhythms and photoperiodic diapause suggest that the circadian clock itself is involved in photoperiodism and that the clock governing photoperiodism is operated by the same machinery in the clock regulating the circadian rhythm.

## Results

### Cuticle deposition rhythm

**Figure 1:**
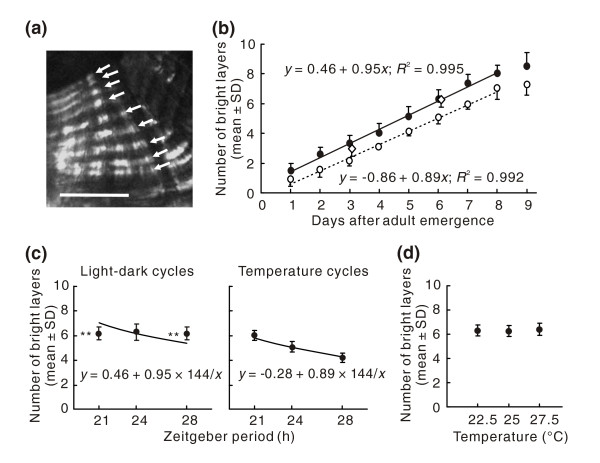
**The properties of the cuticle deposition rhythm in *Riptortus pedestris***. (a) Cross sections of the tibia of the hind leg 20 days after adult emergence under short-day conditions. Alternating double layers are clearly observed in intact individuals. Arrows indicate bright layers. Scale bar: 25 μm. (b) The number of bright layers in the endocuticle under light-dark cycles at a constant temperature (25°C; closed circles), temperature cycles under constant darkness (open circles) and constant darkness at a constant temperature (open diamonds; *N *= 9-16). The solid and broken lines show regression lines calculated from the data from day 1 to day 8. On days 3 and 6 the numbers of bright layers under light-dark cycles were not significantly different from those under constant darkness (*t*-test, *P *> 0.05). The numbers of layers deposited under temperature cycles were one fewer than those under light-dark cycles, suggesting that the rhythm takes a little longer to entrain to temperature cycles. (c) The numbers of bright layers at day 6 (144 h after adult emergence) under light-dark cycles at 25°C (left) and temperature cycles under constant darkness (right; *N *= 11-16). The solid lines show hyperbolae, which are the equations when the rhythm completely entrains to the environmental cycles. The data of *T *= 24 in the left panel were from (b). Asterisks indicate significant differences between the mean number of bright layers and the hypothesized value (*t-*test, ** *P *< 0.01). (d) The number of bright layers on day 6 at 22.5°C, 25°C and 27.5°C under constant darkness. There were no significant differences in the number of bright layers among three temperatures (ANOVA, *P *> 0.05). *N *= 11-13. The data at 25°C are from (b). The *Q*_10 _value was 1.03.

Alternating bright and dark layers were observed in the endocuticle of the hind leg tibia of *R. pedestris *(Figure 1a). First, we confirmed that in *R. pedestris *the cuticle deposition rhythm free-ran under constant conditions (self-sustaining oscillation; Figure 1b), that the number of deposited cuticle layers varied with the given number of temperature cycles (entrainment to environmental cycles; Figure 1c) and that the *Q*_10 _value of the rhythm was close to 1.0 between 22.5°C and 27.5°C (temperature compensation; Figure 1d). Thus, the rhythm exhibited major properties of circadian rhythms, indicating that the cuticle deposition rhythm in *R. pedestris *is regulated by a circadian clock.

### Gene silencing by double-stranded RNA (dsRNA) injection

**Figure 2:**
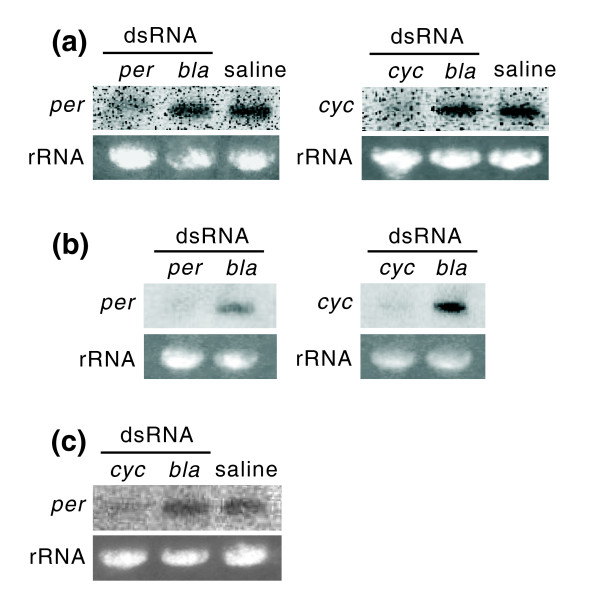
**Gene silencing by *period (per) *and *cycle (cyc) *double-stranded RNA (dsRNA) injection in *Riptortus pedestris***. Northern blotting for *per *(left) and *cyc *(right) was performed using total RNA from the whole bodies of females at Zeitgeber time (ZT) 8 on day 5 (a), or on day 20 (b). Northern blotting for *per *was also performed using total RNA from the heads of *cyc *RNA interference (RNAi) females at ZT8 on day 5 (c). Ribosomal RNAs (rRNAs) are shown as references.

Although gene suppression by dsRNA injection was not obvious in *per *at Zeitgeber time (ZT) 6 (6 h after light-on) and ZT18 on day 5 and in *cyc *at ZT6 on day 5, a clear suppression of target genes was detected in *per *at ZT8 on day 5 and day 20 and in *cyc *at ZT8 and ZT18 on day 5 and at ZT8 on day 20, indicating that RNAi by dsRNA injection is effective in *R. pedestris *(Figure 2a and b and Additional File 1). It should be noted that the expression level of *per *was also reduced in *cyc *RNAi insects (Figure 2c).

### Effects of RNAi on cuticle deposition rhythm

**Figure 3:**
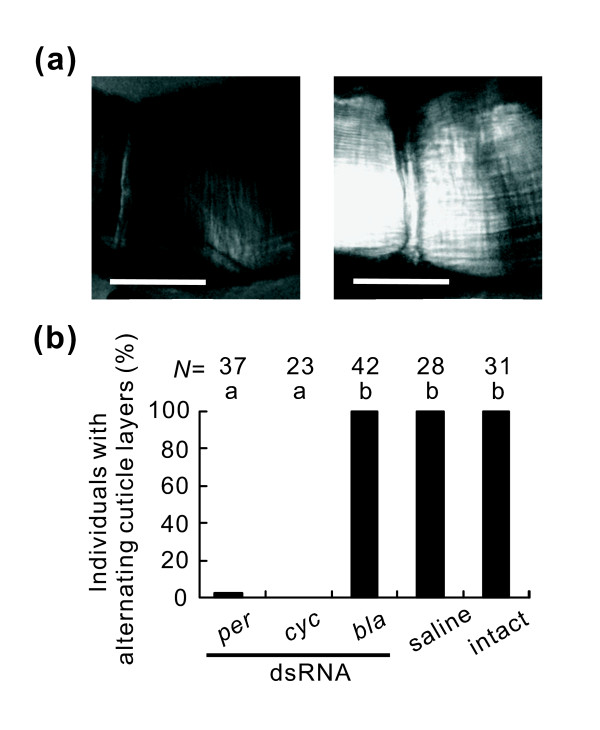
**Effects of *period *(*per) *and *cycle *(*cyc) *RNA interference (RNAi) on the cuticle deposition rhythm in *Riptortus pedestris***. (a) Cross sections of the tibia of the hind leg 20 days after adult emergence under short-day conditions. *per *RNAi (left) produced a single thickened dark layer and *cyc *RNAi (right) produced a single bright layer. Scale bars: 25 μm. (b) Twenty days after injection of *per*, *cyc*, or *β-lactamase *(*bla) *double-stranded RNA (dsRNA) or saline, cuticle layers were observed in the same individuals as shown in Figure 4a. The ordinate shows the percentage of individuals with alternating double layers in the endocuticle. Bars with the same letters are not significantly different (Tukey-type multiple comparisons for proportions, *P *> 0.05).

In *R. pedestris *constantly maintained under short-day conditions, injection of 0.9% NaCl (saline) or control (*β-lactamase*: *bla*) dsRNA did not affect the alternating deposition of bright and dark layers, as in the case of intact insects (Figures 1a and 3b). In contrast, almost all insects injected with *per *dsRNA failed to deposit alternating cuticle layers, resulting in the production of a single thickened dark layer (Figure 3a left and 3b). Injection of *cyc *dsRNA also produced similar results, except that it generated a single bright layer (Figure 3a right and 3b). We also checked the cuticle deposition rhythm in all the individuals used in the following photoperiodic experiments and we obtained similar results irrespective of photoperiodic conditions (Additional File 2).

### Effects of RNAi on diapause

We designed four experimental schedules with different combinations of short-day and long-day conditions before and after adult emergence and injected dsRNAs or saline into females on the day of adult emergence.

**Figure 4:**
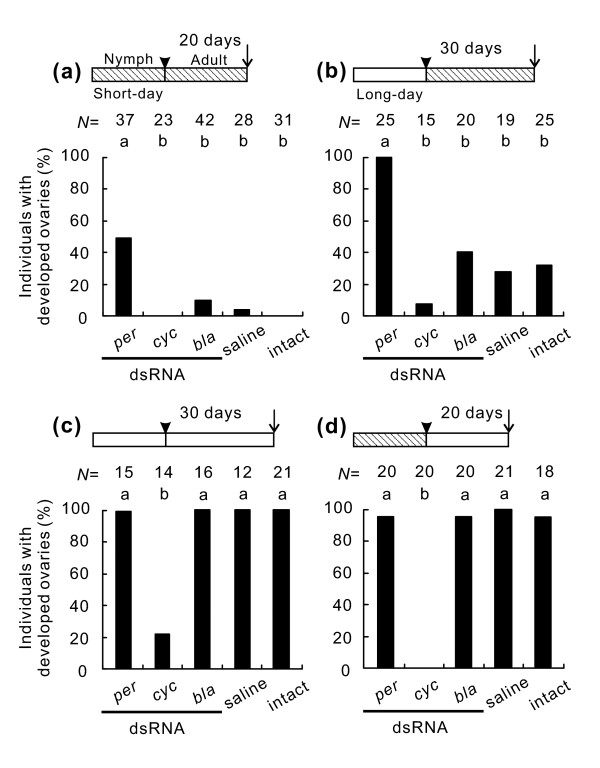
**Effects of *period (per) *and *cycle (cyc) *RNA interference on ovarian development in *Riptortus pedestris***. The experimental schedules are shown as horizontal hatched bars (short-day conditions) or open bars (long-day conditions). Arrowheads show the days of adult emergence and arrows show the days of dissection. Insects were maintained continuously under short-day conditions (a), transferred from long-day to short-day conditions (b), maintained continuously under long-day conditions (c) or transferred from short-day to long-day conditions (d). Bars with the same letters are not significantly different (Tukey-type multiple comparisons for proportions, *P *> 0.05).

In the first experimental schedule, insects were continuously maintained under short-day conditions. Under these conditions, intact females did not develop ovaries and entered diapause in response to short days (Figure 4a) and most females injected with *bla *dsRNA or saline also entered diapause (Figure 4a). None of the individuals injected with *cyc *dsRNA showed any ovarian development (Figure 4a). In contrast, *per *RNAi induced ovarian development in approximately 50% of the females (Figure 4a).

In the second experimental schedule, insects reared under long-day conditions were transferred to short-day conditions on the day of adult emergence. Under these conditions, many intact females and females injected with *bla *dsRNA or saline suppressed ovarian development and entered diapause in response to short-day conditions (Figure 4b). Similarly, most females injected with *cyc *dsRNA suppressed ovarian development. However, mature ovaries were observed in all females injected with *per *dsRNA (Figure 4b).

In the third experimental schedule, insects were continuously maintained under long-day conditions. Under these conditions, all intact females and females injected with *per *dsRNA, *bla *dsRNA, or saline developed ovaries (Figure 4c). However, *cyc *RNAi suppressed ovarian development in approximately 80% of females (Figure 4c).

In the fourth experimental schedule, insects reared under short-day conditions were transferred to long-day conditions on the day of adult emergence. Under these conditions, most intact females and most females injected with *bla *dsRNA or saline developed ovaries (Figure 4d). Similarly, females injected with *per *dsRNA developed ovaries (Figure 4d). In contrast, no females matured their ovaries when *cyc *dsRNA was injected (Figure 4d).

### The expression of juvenile hormone (JH)-regulated genes

In many insects, reproductive diapause is induced by the suppression of JH secretion [30]. Also, in *R. pedestris*, adult diapause is due to the cessation of JH secretion [31]. We were, however, unable to measure the JH concentration in the hemolymph of *R. pedestris *as hemipteran insects possess a novel type of JH [32]. A system that enables the quantification of JH concentrations in hemipterans has yet to be established. As an alternative approach, however, we could estimate the JH concentration by examining the expression of JH-regulated genes; expressions of *cyanoprotein-α *(*CP-α*) and *vitellogenin *(*Vg*) transcripts are induced by JH, whereas *transferrin *(*Tf*) transcript expression is suppressed, in *R. pedestris *[33,34].

**Figure 5:**
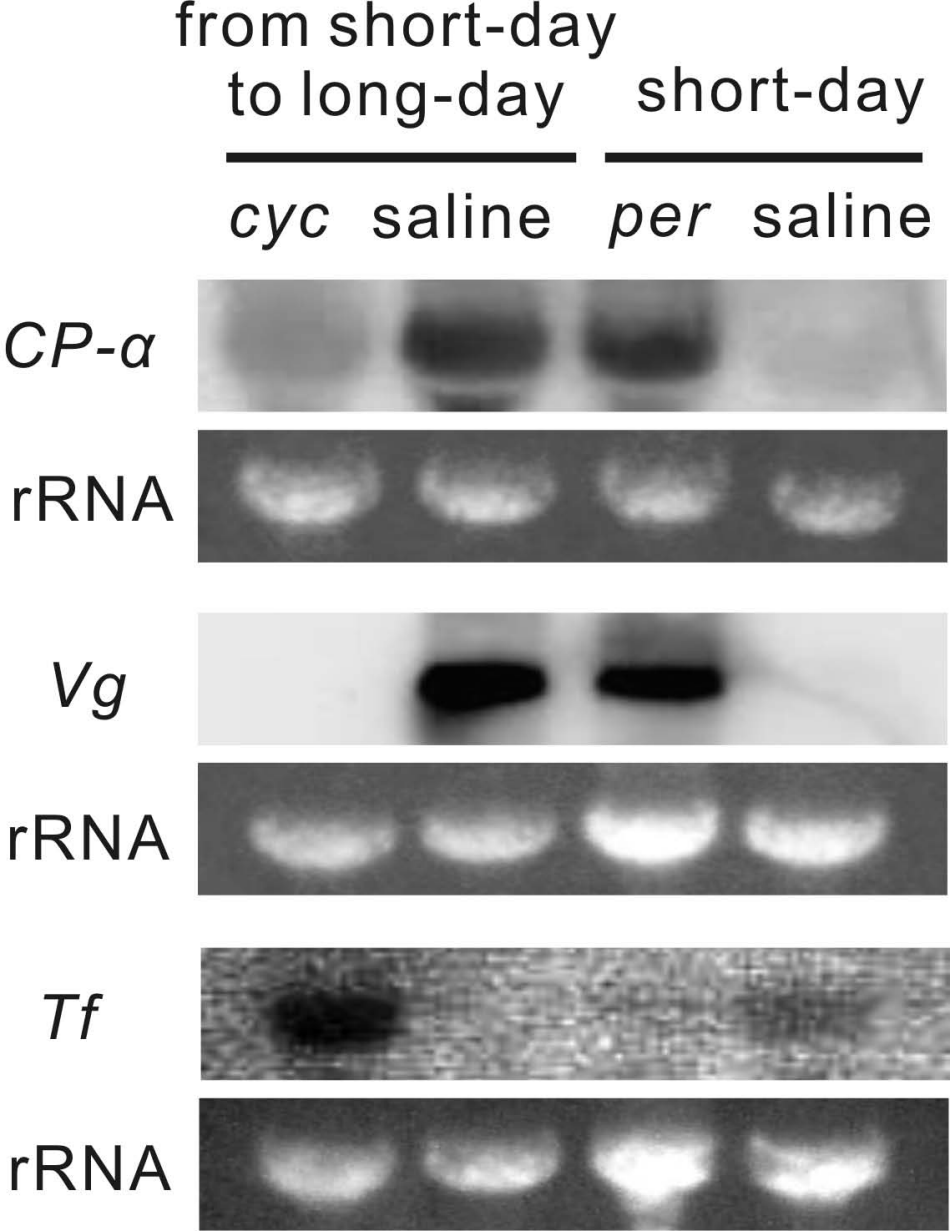
**Effects of *period (per) *and *cycle (cyc) *RNA interference on juvenile hormone-regulated gene expression in *Riptortus pedestris***. Northern blotting for *cyanoprotein-α *(*CP-α*; top), *vitellogenin (Vg*; middle) and *transferrin (Tf*; bottom) was performed with total RNA isolated from the whole bodies of females injected with *per *or *cyc *double-stranded RNA or saline, and transferred from short-day to long-day conditions or maintained continuously under short-day conditions. Ribosomal RNAs (rRNAs) are shown as references.

In this experiment, all insects were reared under short-day conditions. We then transferred some to long-day conditions on the day of adult emergence. *CP-α *and *Vg *transcripts were detected in reproductive females injected with saline and transferred to long-day conditions; they were also detected in reproductive females injected with *per *dsRNA under continuous short-day conditions. However, they were scarcely detectable in non-reproductive females injected with *cyc *dsRNA and transferred to long-day conditions or in non-reproductive females injected with saline under continuous short-day conditions (Figure 5 top and middle). *Tf *transcript was detected in diapause females injected with *cyc *dsRNA and transferred to long-day conditions and in diapause females injected with saline under continuous short-day conditions (Figure 5 bottom). In contrast, the transcript was undetectable in reproductive females injected with saline and transferred to long-day conditions and in reproductive females injected with *per *dsRNA under continuous short-day conditions (Figure 5 bottom). Thus, in reproductive females induced by *per *RNAi, *CP-α *and *Vg *transcripts were clearly expressed, whereas, in contrast, non-reproductive females induced by *cyc *RNAi expressed *Tf *transcript. These results indicate that JH secretion is suppressed in the *cyc *RNAi females, whereas it is induced in the *per *RNAi females.

### JH analogue (JHA) application

**Figure 6:**
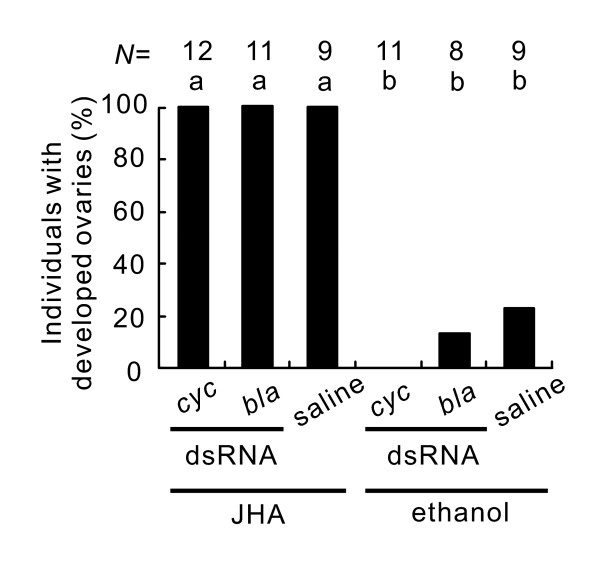
**Effects of juvenile hormone analogue (JHA) application on individuals injected with *cycle *(*cyc) *double-stranded RNA (dsRNA) in *Riptortus pedestris***. dsRNA of *cyc*, *β-lactamase (bla) *or saline was injected into females maintained continuously under short-day conditions. Five days after the injection, JHA or ethanol was applied topically to the abdomen and the reproductive status was checked 10 days after application. Bars with the same letters are not significantly different (Tukey-type multiple comparisons for proportions, *P *> 0.05).

JHA application induced reproduction in females injected with *bla *dsRNA or saline and even in females injected with *cyc *dsRNA. However, the application of a control (ethanol) had little or no effect on the ovarian status of these females (Figure 6). Thus, *cyc *RNAi did not disrupt the process directly involved in ovarian development but did affect some upstream cascade.

## Discussion

In related studies it was shown that in *D. melanogaster *a loss of the cuticle deposition rhythm in clock gene mutants is expressed as an absence of alternating layers in the endocuticle [28]. In this study, RNAi of the circadian clock genes *per *and *cyc *generated cuticle layers in the endocuticle that lacked the distinct alternating pattern. Thus, our results indicate that *per *and *cyc *are core components of the circadian clock generating the cuticle deposition rhythm in *R. pedestris*. It should be noted that RNAi of *per *resulted in the deposition of a single dark layer, whereas that of *cyc *resulted in the deposition of a single bright layer. This indicates that *per *RNAi arrests the clock in a certain phase and thus activates a downstream cascade to produce the dark layer. Similarly, *cyc *RNAi arrests the clock, but in a different phase, thereby activating a distinct downstream cascade to produce the bright layer. In the circadian clock mechanism of *D. melanogaster*, as well as in that of other organisms, PER and CYC are known to play opposite roles - the former is a negative regulator that suppresses CYC activity, whereas the latter is a positive regulator that induces the transcription of *per*, *tim *and many output genes [9]. In our study, the expression level of *per *transcript in *cyc *RNAi insects was low as in *per *RNAi insects, indicating that CYC functions as a positive regulator for *per *transcription in the circadian clock of *R. pedestris *as in other insects.

*R. pedestris *exhibits a clear photoperiodic response; its ovarian development is induced under long-day conditions but is suppressed under short-day conditions (diapause) [21]. This photoperiodic response was disrupted by RNAi of the circadian clock genes: *per *RNAi induced ovarian development even under short-day conditions, whereas *cyc *RNAi suppressed ovarian development even under long-day conditions. These different phenotypes induced by *per *and *cyc *RNAi indicate that the distinct phases at which the circadian clock is arrested by *per *and *cyc *RNAi activate distinct cascades involved in a photoperiodic response, as observed in the cuticle deposition rhythm.

We also verified that JH secretion was suppressed in *cyc *RNAi females, whereas it was induced in *per *RNAi females. In addition, JHA application induced ovarian development in *cyc *RNAi females. We can therefore conclude that these circadian clock genes are involved neither in the process directly regulating ovarian development itself nor in the cascade of events downstream from JH secretion, but that they are involved in an upstream event like a photoperiodic clock [7].

Involvement of circadian clock genes in the photoperiodic responses of insects has been suggested by some authors [15-17]. However, these authors have focused only on single genes. Therefore, these results could be based on pleiotropic effects (non-circadian roles) of the clock genes on diapause itself and not on a malfunction of the circadian clock itself as an integrated physiological function [18-20]. In *R. pedestris*, however, RNAi of *per *and *cyc *induced distinct phenotypes both in the cuticle deposition rhythm and in the photoperiodic response, which suggests that the circadian clock itself, in which PER and CYC function oppositely, regulates not only the circadian rhythm but also the photoperiodic response.

## Conclusions

The cuticle deposition rhythm in *R. pedestris *exhibited major properties of circadian rhythms, suggesting that the rhythm is regulated by a circadian clock. RNAi directed against *per *and *cyc *rendered cuticle deposition arrhythmic and rendered the circadian clock dysfunctional. It should be noted that *per *and *cyc *RNAi produced distinct cuticle layers, and, simultaneously, these RNAi had opposite effects on photoperiodism as well as on JH-regulated gene expression. Based on these results, we suggest that the circadian clock, in which *per *and *cyc *function as opposing regulators, is essential not only for the daily cycles but also for seasonal developmental events.

## Methods

### Insects

Adults of *R. pedestris *were collected in Osaka City (34.6°N, 135.5°E) from July to October in 2007-2009. Their progeny were reared under short-day conditions (12 h light-12 h dark) or long-day conditions (16 h light-8 h dark) at 25°C ± 1°C. Insects were reared individually after adult emergence and only virgin adults were used. All the experiments were performed at 25°C ± 1°C unless otherwise mentioned.

### Observation of cuticle layers

Cuticle layers in the endocuticle were observed in the tibiae of the hind legs of adult insects. The tibiae were cut with a razor blade and were inserted in the pith of an elder. Cross sections that were approximately 50 μm thick were prepared with a simplified microtome. The sections were mounted in distilled water on a slide glass and the bright layers in the endocuticle were counted between crossed polarizers under a light microscope.

### Cuticle deposition rhythm

The cuticle layers in the endocuticle were observed in insects reared from eggs under short-day conditions at 25°C ± 1°C. Some insects reared under the same conditions were transferred to temperature cycles at day 0 and the cuticle layers were observed. Another group of insects reared under light-dark cycles was transferred to constant darkness during the photophase of day 0 and the cuticle layers were observed at the subjective day of day 3 and day 6. Day 0 was defined as the 24-h period after adult emergence.

In order to investigate the entrainability of the cuticle deposition rhythm to environmental cycles, adult insects at day 0 were transferred to light-dark cycles of 10.5 h light-10.5 h dark (the Zeitgeber period, *T *= 21), 12 h light-12 h dark (*T *= 24), or 14 h light-14 h dark (*T *= 28) at 25°C ± 1°C, or temperature cycles of 10.5 h thermophase (30°C ± 1°C) -10.5 h cryophase (20°C ± 1°C) (*T *= 21), 12 h thermophase -12 h cryophase (*T *= 24) or 14 h thermophase -14 h cryophase (*T *= 28) under constant darkness. At the photophase or thermophase of day 6, the cuticle layers were observed.

In order to investigate the temperature compensation of the cuticle deposition rhythm, adult insects reared under short-day conditions at 25°C ± 1°C were transferred to constant darkness at 22.5°C ± 1°C or 27.5°C ± 1°C during the photophase of day 0. The cuticle layers were observed at the subjective day of day 6.

### Reproductive status

The abdomens of insects were dissected in saline under a stereoscopic microscope. Females were classified as being reproductive or non-reproductive (diapause) on the basis of ovarian development as described previously [35] - females with a light-blue yolk deposition in the oocytes were judged to be reproductive and those with no deposition were judged to be in diapause [21].

### RNAi

dsRNAs of *per *and *cyc *were synthesized from the plasmids containing each gene fragment [35]. We also synthesized dsRNA from *bla*, which provides bacteria with ampicillin resistance, using pGEM-T Easy Vector (Promega, Wisconsin, USA); this dsRNA was used as a control. Each plasmid was used as a template for PCR with Pwo DNA Polymerase (Roche, Meylan, France) according to the supplier's instructions. The primers used in the reactions are listed in Additional File 3. dsRNAs were synthesized by using a T7 Ribomax Express RNAi System (Promega) according to the supplier's instructions. Within 24 h after emergence, adults were injected with 1 μg of each dsRNA in 1 μL of saline into the head.

In the present study, we designed four experimental schedules with different combinations of short-day and long-day conditions before and after adult emergence. In the first experimental schedule, insects were continuously maintained under short-day conditions. The reproductive status was checked 20 days later. In the second experimental schedule, insects reared under long-day conditions were transferred to short-day conditions on the day of adult emergence. The reproductive status was checked 30 days later. In the third experimental schedule, insects were continuously maintained under long-day conditions. Thirty days after the injection, their reproductive status was investigated. In the fourth experimental schedule, insects reared under short-day conditions were transferred to long-day conditions on the day of adult emergence. The reproductive status was checked 20 days later. The cuticle layers of the hind leg tibiae in all the insects used for reproductive judgment were also observed at the time of dissection.

### Northern blotting

In order to verify the expression of *per *and *cyc *after each dsRNA injection, northern blotting was performed. Under short-day conditions, the total RNA was isolated from the whole body of a single female at ZT6 and ZT18 on day 5 from the whole bodies of three females at ZT8 on day 5, from heads without the antennae and the rostrum of nine females at ZT8 on day 5 or from the whole bodies of three females at ZT8 on day 20, using Trizol reagent (Invitrogen, California, USA). In order to investigate the expression levels of *per *transcript in insects injected with *cyc *or *bla *dsRNA or saline, the total RNA was isolated from heads without the antennae and the rostrum of seven to 10 females at ZT8 on day 5. RNA probes were synthesized from the linearized plasmid DNA containing the *per *or *cyc *gene fragment. A DNA probe for *β-tubulin*, which was used as a control gene for normalization, was generated from the plasmid containing *β-tubulin *fragment [35].

We also examined the expression of JH-regulated gene, *CP-α*, *Vg*, and *Tf *transcripts in females injected with *per *or *cyc *dsRNAs or saline. On the day of adult emergence, *per *or *cyc *dsRNAs were injected into females reared under short-day conditions from eggs. Females injected with *per *dsRNAs were continuously reared under short-day conditions, and females injected with *cyc *dsRNA were transferred to long-day conditions. The controls were females reared under short-day conditions; they were injected with saline and divided into two groups: one group was transferred to long-day conditions (a control for *cyc *dsRNA injection) and the other was continuously reared under short-day conditions (a control for *per *dsRNA injection). Ten days after the injection, the total RNA was isolated from the whole bodies of three females. Ten micrograms of the total RNA from each female was used in the analysis. DNA probes were synthesized by polymerase chain reaction (PCR) using the primers listed in Additional File 3. RNA and DNA probes were synthesized by using the DIG RNA Labeling kit and PCR DIG probe synthesis kit (Roche), respectively. Hybridization was performed at 68°C for the RNA probes and at 50°C for the DNA probe with DIG Easy Hyb Granules (Roche). Chemiluminescent signals were detected by the Lumino-image analyzer LAS-1000 (Fujifilm, Tokyo, Japan).

### Topical application of JHA

JHA was applied topically to non-reproductive females produced by *cyc *RNAi in order to evaluate their response to JH. JHA is available commercially as Manta^® ^(Otsuka Chemicals Co, Osaka, Japan) which is an ethanol solution of methoprene. Females reared under short-day conditions were injected with *cyc *or *bla *dsRNA or saline on the day of adult emergence. Five days after injection, 2.5 μg JHA in 2 μL ethanol was applied topically to the abdomen of the females. In the females who were used as controls, only 2 μL ethanol was applied. Ten days after application, the reproductive status was examined.

## Abbreviations

*bla: β-lactamase*; *CP-α: cyanoprotein-α*; *cyc: cycle*; dsRNA: double-stranded RNA; JH: juvenile hormone; JHA: JH analogue; *per: period*; PCR: polymerase chain reaction; RNAi: RNA interference; *Tf: transferrin*; *tim: timeless*; *Vg: vitellogenin*; ZT: Zeitgeber time.

## Authors' contributions

HN and SGG designed the research. TI performed the research. TI, SGG, SIT and HN analysed the data. TI, HN and SGG wrote the paper. All authors read and approved the final manuscript.

## Supplementary Material

Additional file 1 **Gene silencing by double-stranded RNA (dsRNA) injection in *Riptortus pedestris***. (A) Northern blotting for *period *(*per*; left) and *cycle *(*cyc; *right) was performed using total RNA from the heads of nine females at Zeitgeber time (ZT) 8 on day 5. Ribosomal RNAs (rRNAs) are shown as references. (B) Relative transcript levels (the intensity ratios to *β-tubulin*) of *per *(left) and *cyc *(right) at ZT6 and 18 on day 5 quantified by northern blotting using total RNA from the whole bodies. The standard curve methodology was adopted. The levels of *per *in females injected with *per *dsRNA were not significantly different from those in females injected with *β-lactamase *(*bla) *dsRNA at both ZT6 and 18 (*t- *test, *P *> 0.05). The level of *cyc *in females injected with *cyc *dsRNA was significantly lower than that in females injected with *bla *dsRNA at ZT6 (*t-*test, *P *< 0.05), but not at ZT18 (*t-*test, *P *> 0.05). The highest value in each experiment was set at 1.0. Each plot represents a total RNA extracted from the whole body of a single female (*N *= 3) for each ZT and treatment. Click here for file

Additional file 2 **Effects of *period *(*per) *and *cycle *(*cyc) *RNA interference on the cuticle deposition rhythm in *Riptortus pedestris***. After injection of *per*, *cyc*, *β-lactamase (bla) *double-stranded RNA (dsRNA) or saline on the day of adult emergence, cuticle layers were observed between crossed polarizers under a light microscope. The ordinate shows the percentage of individuals with alternating double layers in the endocuticle. The experimental schedules are shown as horizontal hatched bars (short-day conditions) or open bars (long-day conditions). Arrowheads show the day of adult emergence and arrows show the day of the observation. Insects were transferred from long-day to short-day conditions (A), maintained continuously under long-day conditions (B), or transferred from short-day to long-day conditions (C). The bars with the same letters are not significantly different (Tukey-type multiple comparisons for proportions, *P *> 0.05). Click here for file

Additional file 3 **Sequences of primers**. Click here for file
