# Supplementary figures and images for: Photoperiodic diapause under the control of circadian clock genes in an insect
Source: BMC Biol. 2010 Sep 3;8:116. doi: 10.1186/1741-7007-8-116 (PMC2942818; doi:10.1186/1741-7007-8-116)

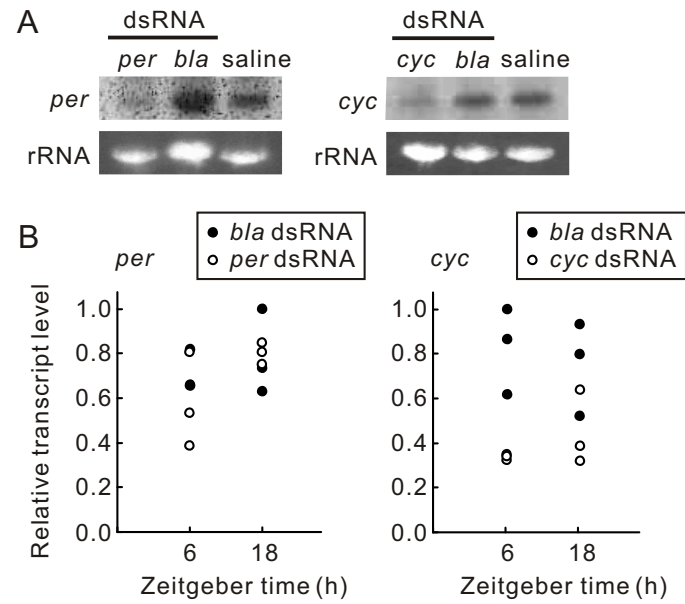

Supplement: Additional file 1 — Gene silencing by double-stranded RNA (dsRNA) injection in Riptortus pedestris. (A) Northern blotting for period (per; left) and cycle (cyc; right) was performed using total RNA from the heads of nine females at Zeitgeber time (ZT) 8 on day 5. Ribosomal RNAs (rRNAs) are shown as references. (B) Relative transcript levels (the intensity ratios to β-tubulin) of per (left) and cyc (right) at ZT6 and 18 on day 5 quantified by northern blotting using total RNA from the whole bodies. The standard curve methodology was adopted. The levels of per in females injected with per dsRNA were not significantly different from those in females injected with β-lactamase (bla) dsRNA at both ZT6 and 18 (t- test, P > 0.05). The level of cyc in females injected with cyc dsRNA was significantly lower than that in females injected with bla dsRNA at ZT6 (t-test, P < 0.05), but not at ZT18 (t-test, P > 0.05). The highest value in each experiment was set at 1.0. Each plot represents a total RNA extracted from the whole body of a single female (N = 3) for each ZT and treatment. [file 1741-7007-8-116-S1.pdf]

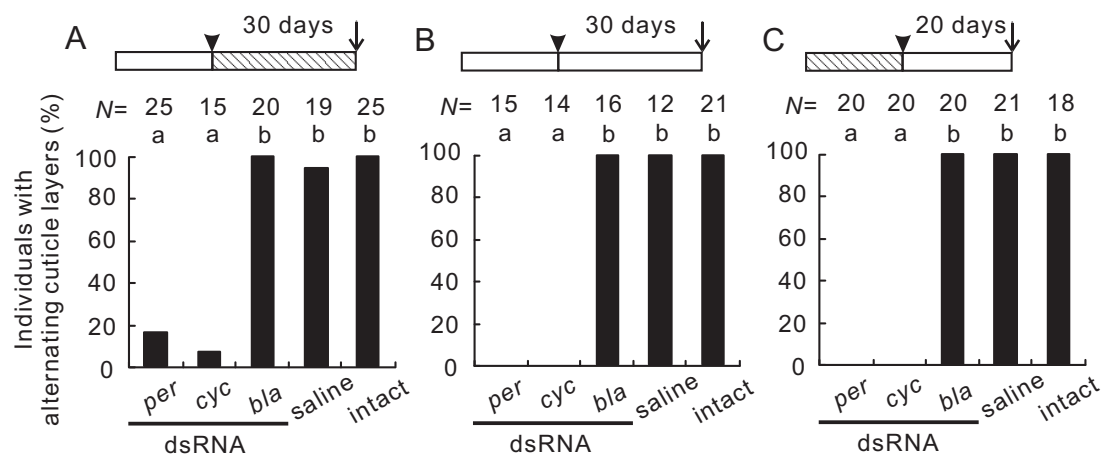

Supplement: Additional file 2 — Effects of period (per) and cycle (cyc) RNA interference on the cuticle deposition rhythm in Riptortus pedestris. After injection of per, cyc, β-lactamase (bla) double-stranded RNA (dsRNA) or saline on the day of adult emergence, cuticle layers were observed between crossed polarizers under a light microscope. The ordinate shows the percentage of individuals with alternating double layers in the endocuticle. The experimental schedules are shown as horizontal hatched bars (short-day conditions) or open bars (long-day conditions). Arrowheads show the day of adult emergence and arrows show the day of the observation. Insects were transferred from long-day to short-day conditions (A), maintained continuously under long-day conditions (B), or transferred from short-day to long-day conditions (C). The bars with the same letters are not significantly different (Tukey-type multiple comparisons for proportions, P > 0.05). [file 1741-7007-8-116-S2.pdf]
