# Supplementary material for: Photoperiodic diapause under the control of circadian clock genes in an insect
Source: BMC Biol. 2010 Sep 3;8:116. doi: 10.1186/1741-7007-8-116 (PMC2942818; doi:10.1186/1741-7007-8-116)
Supplement: Additional file 3 — Sequences of primers. [file 1741-7007-8-116-S3.pdf]

Additional File 3

	Oligonucleotides	Sequences (5' >> 3')
For dsRNA synthesis		
per	per10-F	GGG GAA GAT TTC TCC CGT AG
	per21T7-R	TAA TAC GAC TCA ATC TAG GGA ACG TAG GGC ATT TGC TGT
	per10T7-F	TAA TAC GAC TCA ATC TAG GGG GGA AGA TTT CTC CCG TAG
	per21-R	GAA CGT AGG GCA TTT GCT GT
cyc	cyc5-F	GGC GCT AGG AGA TCC TTC TT
	cyc5T7-R	TAA TAC GAC TCA ATC TAG GCT CTG GAG GGC AGC TTT ATG
	cyc5T7-F	TAA TAC GAC TCA ATC TAG GGG CGC TAG GAG ATC CTT CTT
	cyc5-R	CTC TGG AGG GCA GCT TTA TG
bla	pGBetalacm-F1	TCG CCG CAT ACA CTA TTC TC
	pGBetalacmT7-R1	TAA TAC GAC TCA ATC TAG GGA GAC CAC GTA CGA TAC GGG AGG GCT TAC
	pGBetalacmT7-F1	TAA TAC GAC TCA ATC TAG GGA GAC CAC GTC GCC GCA TAC ACT ATT CTC
	pGBetalacm-R1	TAC GAT ACG GGA GGG CTT AC
For probe synthesis		
CP-α	cpa1-F	CTG AGC TTT TCA AAC CAC AAA
	cpa1-R	TGG TCA ACG ATA GAC ACA GCA
Vg	vg1-F	AGC GAA CTT CAG GCT GGA TA
	vg1-R	TGA GCA GTG TTT CCA AGT GC
Tf	transF	GGG AAT TCT TGG ACC ACT GA
	transR	GGC ATC GGG ATA GTC ACA CT
